# Circulating Levels of Inflammatory Proteins and Survival in Patients with Gallbladder Cancer

**DOI:** 10.1038/s41598-018-23848-8

**Published:** 2018-04-04

**Authors:** Zhiwei Liu, Troy J. Kemp, Yu-Tang Gao, Amanda Corbel, Emma E. McGee, Juan Carlos Roa, Bingsheng Wang, Juan Carlos Araya, Ming-Chang Shen, Asif Rashid, Ann W. Hsing, Allan Hildesheim, Catterina Ferreccio, Ruth M. Pfeiffer, Ligia A. Pinto, Jill Koshiol

**Affiliations:** 1 Infections and Immunoepidemiology Branch of the Division of Cancer Epidemiology and Genetics, National Cancer Institute, Bethesda, Maryland USA; 2 HPV Immunology Laboratory, Frederick National Laboratory for Cancer Research, Leidos, Biomedical Research, Inc, Frederick, MD USA; 3 Department of Epidemiology, Shanghai Cancer Institute, Shanghai, China; 4 School of Medicine, Pontificia Universidad Católica de Chile, Santiago, Chile; 5 Advanced Center for Chronic Diseases (ACCDiS), FONDAP, Santiago, Chile; 6 Department of General Surgery, Zhongshan Hospital, School of Medicine, Fudan University, Shanghai, China; 7 Hospital Dr. Hernan Henríquez Aravena, Temuco, Chile; 8 Anatomic Pathology Department, Medicine Faculty, Universidad de La Frontera, Temuco, Chile; 9 Department of Pathology, Shanghai Cancer Center, Fudan University, Shanghai, China; 10 Department of Pathology, The University of Texas MD Anderson Cancer Center, Houston, TX USA; 11 Stanford Cancer Institute, Palo Alto, CA USA; 12 Department of Health Research and Policy, Stanford School of Medicine, Palo Alto, CA USA; 13 Biostatistics Branch, Division of Cancer Epidemiology and Genetics, National Cancer Institute, Bethesda, MD USA

## Abstract

Although inflammation is central to gallbladder cancer (GBC) development and proliferation, no study has systematically investigated circulating inflammatory proteins and patient survival. We aimed to examine whether the circulating levels of inflammatory proteins is associated with all-cause mortality among such patients. We recruited 134 patients with newly diagnosed with GBC from 1997 to 2001 in a population-based study in Shanghai and an independent set of 35 patients from 2012 to 2013 in Chile. Cox proportional hazards regression models adjusted for covariates were used to evaluate the hazard ratios (HRs) for death by serum levels of 49 inflammatory proteins (quartiles). Of 49 evaluable proteins, eight were significantly associated with overall survival. Seven were associated with a poorer survival, while the highest levels of tumor necrosis factor-related apoptosis-inducing ligand (TRAIL) were associated with an increase in survival (HR = 0.26, 95% CI = 0.14, 0.47). No substantial difference in the magnitude of the association was observed between early- and late-stages of GBC. Of seven proteins, five were validated in the patients from Chile. Reducing inflammation and targeting pathways associated with increased survival might improve GBC outcomes. The potential for using a TRAIL-related anticancer drug for GBC treatment merits further investigation.

## Introduction

Gallbladder cancer (GBC) is one of the most common and aggressive cancers of the biliary tract^1^. Five-year survival rates range from 5% to 20%^2–4^. Among patients diagnosed with localized tumors, the 5-year survival rate can extend to >40%^4^. Complete surgical resection is the only curative therapy available; however, most patients (>90%) are diagnosed with late-stage or metastatic diseases that are not amenable to curative therapy^5,6^. Treatments to improve survival in these patients are urgently needed.

Factors associated with gallbladder cancer survival are poorly understood. Other than disease stage at presentation, few patient characteristics have been identified that predict survival time in GBC patients^7–9^. Better understanding the factors that are related to survival may assist risk stratification and development of targeted therapies^10^. For example, studies have consistently reported that aspirin and/or other nonsteroidal anti-inflammatory drug use is associated with an improvement in survival among patients with colorectal cancer^11^, and aspirin use has been proposed as an adjuvant therapy for colorectal cancer^12^.

The link between inflammation and gallbladder cancer risk is well established^13–17^, largely due to the fact that gallstones, which create extensive inflammation in the gallbladder, are the central risk factor for GBC^18^. Other potential risk factors are also likely act through inflammatory pathways, including diabetes, obesity, infections (e.g. *Helicobacter* species), metabolic syndrome, and other inflammation-related conditions^13–17^. However, the role of inflammation in the prognosis of GBC patients is largely unknown. Previous studies have reported associations with survival for only a limited number of inflammatory proteins representing a small subset of the inflammation cascade, such as C-reactive protein (CRP)^19,20^, interleukin 6 (IL-6)^21^, and 17 (IL-17)^22^. However, previous studies are limited by single-center study design or lack of replication.

Therefore, to systematically investigate whether circulating inflammatory proteins are related to GBC prognosis, we evaluated the association between circulating inflammatory proteins and overall survival among patients with GBC from Shanghai, China, where the age-standardized incidence rates of GBC are moderately high: ~3 per 100,000 person-years^23^, as opposed to ~1 per 100,000 person-years in the US^24^. We then verified the findings among patients from Chile, where the age-standardized incidence rates are among the highest in the world, reaching 8.6 and 24.3 per 100,000 person-years in males and females, respectively^3^.

## Methods

### Study population

Details of the Shanghai Biliary Cancer Study have been published previously^25^. Briefly, patients aged 35 to 74 years who were permanent residents of urban Shanghai with newly diagnosed primary biliary tract cancer (ICD 9 = 156) were identified through a rapid-reporting system established between the Shanghai Cancer Institute and 42 collaborating hospitals in 10 urban districts of Shanghai. Between June 1997 and May 2001, a total of 368 GBC cases were recruited, representing over 90% of all eligible GBC cases.

Because cholecystectomy (as a major treatment option) may affect the level of circulating inflammation proteins, of the 368 patients with GBC recruited, serum samples from 144 were tested for inflammatory proteins, including those who did not have cholecystectomy, or had serum collected prior to cholecystectomy. Vital status of all patients was identified through linkage to the Population Death Register until March 2003. Ten patients were excluded because of insufficient clinical (N = 2) or follow-up (N = 8) data, leaving 134 patients for analysis. We compared characteristics of patients who were included in this study versus those who were not included. No appreciable difference was noticed (Supplementary Table 1).

We replicated the results from the Shanghai study in patients with GBC from the Chile Gallbladder Cancer Study, a study from which we had data on circulating inflammatory proteins and survival. Details about the Chile Gallbladder Cancer Study have been published^14,26^. Briefly, between April 2012 and August 2013, we recruited 52 GBC cases newly diagnosed at cancer referral hospitals in Santiago, Concepción, and Temuco, with a participation rate of 85%. Thirty-five GBC cases with available serum samples and inflammation protein data were included in the validation study. Vital status of all GBC cases was identified through linkage to the Population Death Register until March 2017.

For both studies, written informed consent was obtained for biospecimen and questionnaire data collection for all participants. The Shanghai study was approved by the Institutional Review Boards of the National Cancer Institute and the Shanghai Cancer Institute. The Chile study was approved by Institutional Review Boards at the National Cancer Institute, Pontificia Universidad Católica de Chile, and the Chilean Ministry of Health. All methods were performed in accordance with the relevant guidelines and regulations.

### Laboratory Methods

All blood samples were continuously stored in freezers at −70 °C. In order to screen a large number of inflammation proteins, we tested these stored specimens using the recently-developed Luminex bead-based assay (EMD Millipore, Billerica, MA, USA) and, for acute phase markers, Meso Scale Discovery (MSD) Human Vascular Injury II kit (Meso Scale Diagnostics LLC, Rockville, MD, USA)^14,15^. A total of 68 inflammatory proteins covering several key components of inflammation, including acute-phase proteins, pro- and anti-inflammatory cytokines, chemokines, growth factors, and angiogenesis factors were included. Details about these assays have been published^14,15^, and the selection of the proteins is described in detail in Supplementary Materials. Briefly, we excluded 19 proteins with overall coefficients of variations >30%, intraclass correlation coefficients <0.75, and/or duplicated measurements. Thus, the final analysis of the Shanghai study included 49 evaluable proteins.

### Statistical analyses

We first identified inflammatory proteins that were associated with overall survival in the Shanghai study. Since subset of the samples from the Shanghai study were tested with a different lot of kits as described in the Supplemental Materials, study-specific cutpoints were used to generate categories. Because all proteins were detectable in all GBC cases, they were grouped into quartiles. Overall survival time was calculated from the date of sample collection (for 1 subject without recorded date of sample collection, date of diagnosis was used) until the date of death or last follow-up if a participant was still alive. We used Cox proportional hazards regression models to estimate hazard ratios (HRs) and 95% CIs with overall survival. Age group at diagnosis (≤54, 55–65, or ≥66 years), sex, clinical stage (early [i.e., resectable, local, stages 1 and 2] or late [i.e., stages 3 and 4]), and whether the patient received a cholecystectomy were included in the minimally adjusted models because these variables have been previously reported to be associated with GBC survival^6,8,9^.

In addition, we conducted stepwise linear regression to determine whether inflammation marker levels were associated with: education (none/primary, junior middle, senior middle, some college), ever drinking, ever smoking, self-reported body mass index (underweight, normal weight, overweight, obese), regular aspirin use (one year prior to interview), fasting status (fasting/not fasting), and history of hypertension, diabetes, kidney or bladder infection, tuberculosis, chronic gastritis, gastric ulcer, duodenal ulcer, appendicitis, coronary heart disease, and cholecystitis. For variables that were associated with proteins in linear regression, we considered whether the additionally included variables would change the minimally adjusted HR of overall survival for a one-category increase in inflammatory protein level by more than 10%. Based on this criterion, no additional variables were included in the adjusted models. The proportionality of hazards assumption was examined by evaluating a time-dependent variable, which was the cross-product of treating marker categories as ordinal variables and time. No violation was observed after accounting for multiple testing. All models were stratified by lot.

Due to the potentially strong correlation between levels of inflammatory proteins and stage at diagnosis, for the Shanghai study we estimated median survival time adjusted for clinical stage using direct adjusted survival estimation with the mean of covariates method. The survival estimates were weighted by the overall proportion of patients with early- vs. late-stage GBC^27,28^.

To test whether the association between proteins and overall survival differed by clinical stage or cholecystectomy, stratified analyses were performed. Heterogeneity across stratum was evaluated using the likelihood ratio test with and without interaction terms in the Cox proportional hazards regression model. At the time of analysis, we identified 4 samples that were collected after cholecystectomy. Thus, we also conducted analyses excluding these samples (N = 130). Growth of tumors after diagnosis could have affected levels. To the authors’ knowledge, no information on the growth rate of gallbladder cancer has yet been published. Using the minimum doubling time reported for hepatocellular carcinoma as an example (doubling time ranging from 30 to 600 days)^29,30^, we thus assumed the doubling time could be 30 days and performed a sensitivity analysis focusing on samples collected no more than 1 month after diagnosis (N = 108). We also conducted a backward step-wise regression model including all selected inflammatory proteins to determine the proteins independently associated with overall survival.

Inflammatory proteins associated with overall survival in the Shanghai study were also assessed in the Chile study^14^. The information on clinical stage was not complete for patients from Chile, so we used Cox proportional hazards regression models adjusted for sex and age at diagnosis. We did not adjust for cholecystectomy because 94% of patients (33 of 35) had surgical resection.

Statistical analyses were performed using SAS software, version 9.4 (SAS Institute, Cary, NC, USA) and R statistical software (http://www.r-project.org/). The statistical tests were two tailed with α = 0.05. For Shanghai study, Bonferroni corrected *P* values (0.05/49 = 0.001) were calculated to account for multiple testing.

## Results

**Figure 1:**
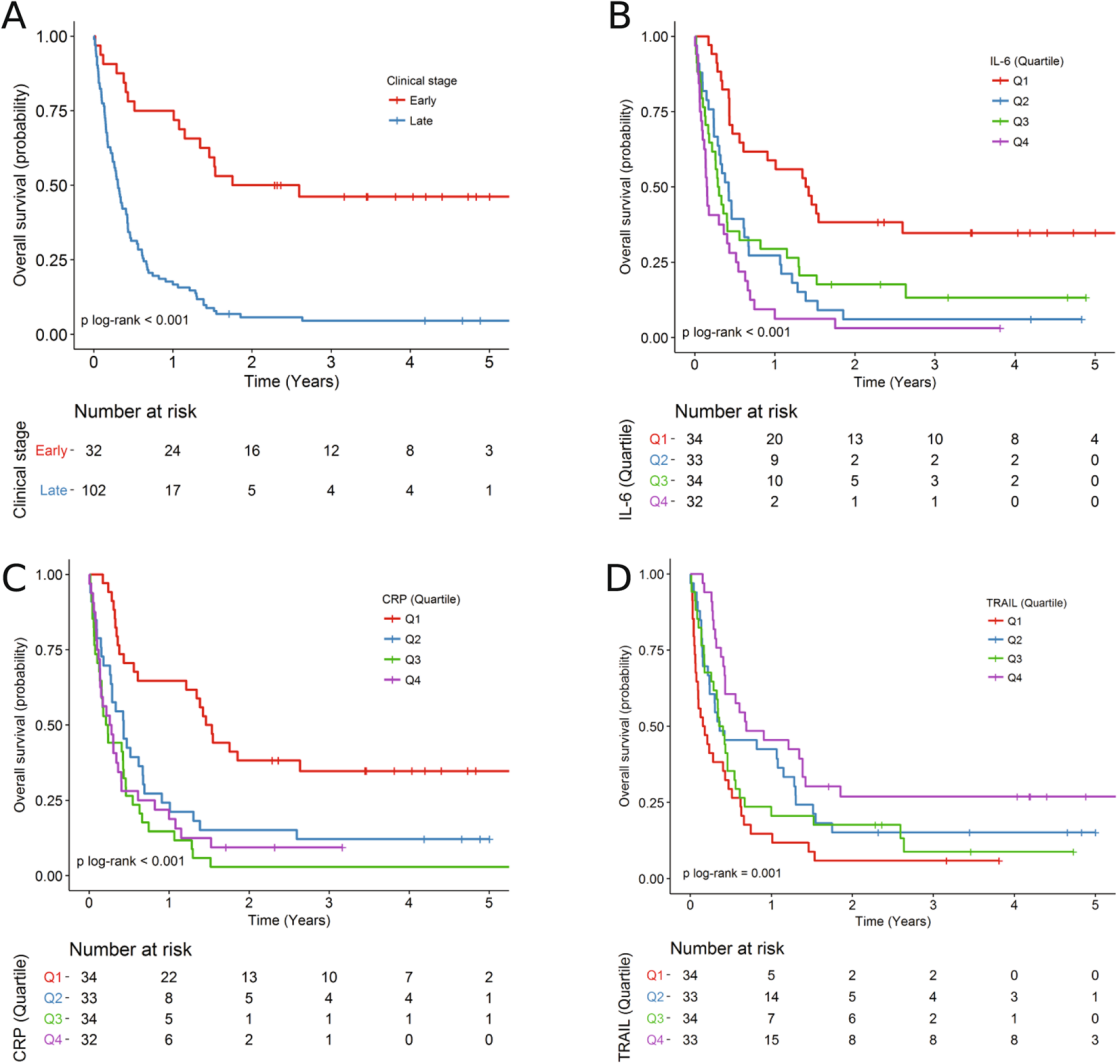
Kaplan-Meier survival estimates for patients with GBC. Overall survival curves of evaluated patients stratified by (**A**) clinical stage, (**B**) circulating IL-6 levels (quartile), (**C**) circulating CRP levels (quartile), and (**D**) circulating TRAIL levels (quartile). Log-rank test used to calculate *P* values.

In the Shanghai study, unadjusted median survival was 5.1 months (interquartile range [IQR]: 1.8–15.9 months) for all patients, with 24.6 months (IQR: 9.3–47.8 months) for patients with early-stage and 3.7 months (IQR: 1.6–7.7 months) for patients with late stages (Table 1 and Fig. 1A). Patients with early-stage were more likely to have cholecystectomy. For all other patient characteristics, we found no appreciable differences between early- and late-stages. By the end of follow-up, 114 patients (85.1%) were deceased. Characteristics of the 35 patients from the Chile study have been published previously^14^. Unadjusted median survival was 9.9 months (IQR: 0.6–11.2 months).

**Table 1:** Characteristics of Patients with Gallbladder Cancer in Shanghai^a^.

	Clinical stage		*P* value
	Early-stage (N = 32, %)	Late-stage (N = 102, %)	
**Survival (months)**			<0.001^b^
Median (interquartile range)	24.6 (9.3–47.8)	3.7 (1.6–7.7)	
**Age, years**			0.754
≤54	4 (12.5)	9 (8.8)	
55–65	9 (28.1)	33 (32.4)	
≥66	19 (59.4)	60 (58.8)	
**Sex**			0.653
Male	9 (28.1)	33 (32.3)	
Female	23 (71.9)	69 (67.7)	
**Education**			0.749
None/Primary	18 (56.2)	56 (54.9)	
Jr. Middle	7 (21.9)	26 (25.5)	
Sr. Middle	2 (6.3)	10 (9.8)	
Some college	5 (15.6)	10 (9.8)	
**Self-reported body mass index**			0.683
Underweight: < 18.5	2 (6.2)	9 (8.8)	
Normal: 18.5–24.9	15 (46.9)	55 (53.9)	
Overweight: 25–29.9	14 (43.8)	32 (31.4)	
Obese: ≥30.0	1 (3.1)	6 (5.9)	
**Regular Aspirin use (one year prior to interview)**			0.328
No	0 (0.0)	4 (4.0)	
Yes	32 (100.0)	97 (96.0)	
Missing	0	1	
**Ever smoker**			0.124
No	26 (81.3)	66 (65.4)	
Yes	6 (18.7)	35 (34.7)	
Missing	0	1	
**Ever drinker**			0.791
No	26 (81.2)	85 (83.3)	
Yes	6 (18.8)	17 (16.7)	
**Gallstone**			0.454
No	8 (25.0)	19 (18.6)	
Yes	24 (75.0)	83 (81.4)	
**Cholecystitis**			0.667
No	23 (71.9)	67 (65.7)	
Yes	9 (28.1)	35 (34.3)	
**Cholecystectomy**			0.043
No	8 (25.0)	46 (45.1)	
Yes	24 (75.0)	56 (54.9)	

^a^Continuous variables reported as median (interquartile range) and categorical variables reported as No. (%). ^b^*P* value was determined using a log-rank test. Other *P* values were determined using a chi-square test or Fisher’s exact test (when the number of subjects in a cell was <5).

Of the 49 inflammatory proteins in the Shanghai study, seven were statistically significantly associated with overall survival in patients with GBC in multivariable adjusted models (using *P*_trend_ < 0.001, Bonferroni correction, Table 2 and Supplementary Table 2). Another one was at borderline significance (*P*_trend_ = 0.001). These proteins included chemokines (chemokine [C-C motif] ligand 19 [CCL19] and 20 [CCL20]), an acute-phase protein (CRP), proinflammatory cytokines (soluble tumor necrosis factor receptor 1 [sTNFRI] and 2 [sTNFRII], and IL-6), growth factors (soluble vascular endothelial growth factor receptor 3 [sVEGFR3], and tumor necrosis factor-related apoptosis-inducing ligand (TRAIL). Kaplan-Meier survival estimates for 2 frequently evaluated proteins (IL-6 and CRP), and one protein (TRAIL) were shown in Fig. 1B–D, and others in Supplementary Figs 1–5. Elevated levels of seven proteins were associated with a poorer survival in patients with GBC, with adjusted HRs (comparing Quartile 4 [Q4] vs 1 [Q1]) ranging from 2.49 (95% CI = 1.41, 4.41) for chemokine (C-C motif) ligand 20 (CCL20) to 3.77 (95% CI = 1.98, 7.19) for tumor necrosis factor-alpha receptor II (sTNFRII). The difference in stage-adjusted median survival time between Q4 and Q1 ranged from 4.7 to 10.1 months (Table 2). In contrast, elevated levels of TRAIL were associated with an increased survival (HR for Q4 vs Q1 = 0.26; 95% CI = 0.14, 0.47, *P*_trend_ across marker categories = 8.3 × 10^−5^). The median stage-adjusted survival time was 7.9 months longer in patients with the highest levels of TRAIL compared to those with the lowest levels (Table 2).

**Table 2:** Associations between inflammatory proteins and mortality among patients with gallbladder cancer in Shanghai.

Inflammatory proteins	No. of patients	No. of deaths	Adjusted HRs	Adjusted median survival (months)^b^
		(% of patients)	(95% CIs)^a^	
**CCL19**				
Q1	34	22 (64.7)	1.00	11.1
Q2	33	29 (87.9)	1.49 (0.82, 2.72)	7.7
Q3	34	32 (94.1)	2.08 (1.13, 3.82)	3.9
Q4	33	31 (93.9)	2.86 (1.58, 5.17)	3.1
*P* trend^c^			2.2 × 10^−4^	
**CCL20**				
Q1	34	25 (73.5)	1.00	8.2
Q2	33	28 (84.8)	1.82 (1.05, 3.17)	5.2
Q3	34	30 (88.2)	2.62 (1.50, 4.58)	3.9
Q4	33	31 (93.9)	2.49 (1.41, 4.41)	3.1
*P* trend^c^			6.3 × 10^−4^	
**CRP**				
Q1	34	22 (64.7)	1.00	10.0
Q2	33	29 (87.9)	1.84 (1.02, 3.34)	5.2
Q3	34	33 (97.1)	2.51 (1.36, 4.62)	3.7
Q4	32	29 (90.6)	2.90 (1.58, 5.32)	2.9
*P* trend^c^			3.3 × 10^−4^	
**IL-6**				
Q1	34	22 (64.7)	1.00	8.2
Q2	33	31 (93.9)	1.78 (0.98, 3.21)	5.2
Q3	34	29 (85.3)	1.72 (0.94, 3.12)	3.9
Q4	32	31 (96.9)	3.02 (1.66, 5.48)	3.1
*P* trend^c^			7.0 × 10^−4^	
**sTNFRI**				
Q1	34	26 (76.5)	1.00	10.0
Q2	33	24 (72.7)	1.35 (0.76, 2.39)	5.1
Q3	34	34 (100.0)	3.82 (2.22, 6.57)	3.5
Q4	31	29 (93.5)	2.56 (1.43, 4.59)	2.6
*P trend* ^c^			2.9 × 10^−5^	
**sTNFRII**				
Q1	34	23 (67.6)	1.00	12.3
Q2	33	29 (87.9)	1.36 (0.75, 2.49)	7.6
Q3	34	31 (91.2)	2.40 (1.31, 4.38)	3.5
Q4	31	30 (96.8)	3.77 (1.98, 7.19)	2.2
*P trend*^c^			7.9 × 10^−6^	
**sVEGFR3**				
Q1	34	26 (76.5)	1.00	8.1
Q2	33	27 (81.8)	2.59 (1.44, 4.66)	5.0
Q3	34	33 (97.1)	2.81 (1.61, 4.90)	3.7
Q4	31	27 (87.1)	2.56 (1.40, 4.67)	3.2
*P trend*^c^			1.3 × 10^−4^	
**TRAIL**				
Q1	34	32 (94.1)	1.00	2.9
Q2	33	28 (84.8)	0.46 (0.27, 0.80)	3.7
Q3	34	30 (88.2)	0.63 (0.37, 1.08)	5.2
Q4	33	24 (72.7)	0.26 (0.14, 0.47)	10.0
*P trend*^c^			8.3 × 10^−5^	

^a^Adjusted for age groups (≤54, 55–65, or ≥66 years), sex, clinical stage (early or late), and ever had cholecystectomy (yes/no), stratified by lot. ^b^Adjusted for clinical stage (early or late). ^c^Two-sided P values for trend across marker categories were assessed with the Wald test using categorical values of the proteins with 1 degree of freedom.

**Figure 2:**
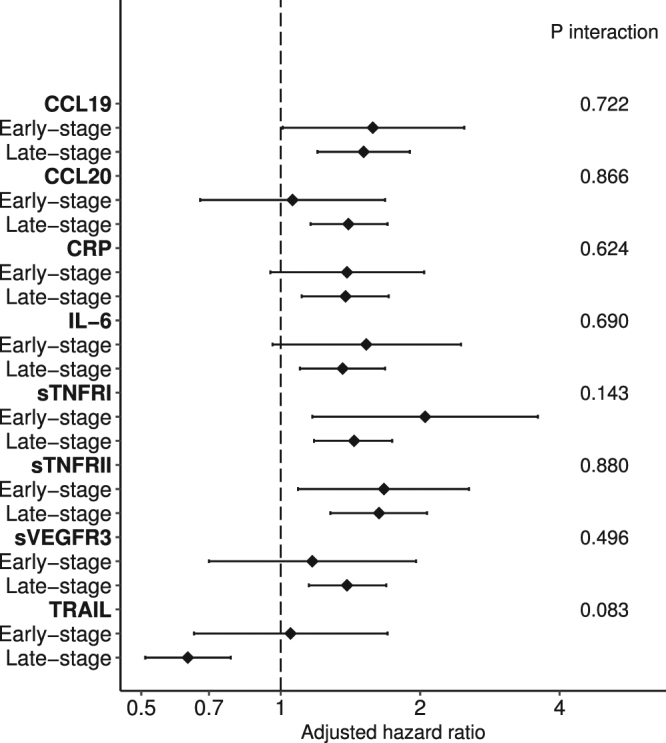
Associations between inflammatory proteins and mortality among patients with gallbladder cancer in Shanghai, stratified by clinical stage at diagnosis (early- vs late-stage).

Of the eight proteins associated with survival, five (CCL19, CCL20, sTNFRI, sVEGFR3, and TRAIL) were not correlated with clinical stage **(**Supplementary Table 3**)**. Notably, in patients with early-stage GBC, poorer survival was associated with elevated levels of CCL19 (HR for one category increase = 1.58, 95% CI = 1.01, 2.48), CRP (HR = 1.39, 95% CI = 0.95, 2.04), IL-6 (HR = 1.53, 95% CI = 0.96, 2.45), sTNFRI (HR = 2.05, 95% CI = 1.17, 3.59), and sTNFRII (HR = 1.67, 95% CI = 1.09, 2.55, Fig. 2). In contrast, elevated levels of TRAIL were associated with an increased overall survival among patients with late-stage GBC (HR = 0.63, 95% CI = 0.51, 0.77) but not early-stage GBC (HR = 1.05, 95% CI = 0.65, 1.71). However, in multivariable adjusted survival models, no statistically significant interaction was observed between marker levels and either clinical stage (early- vs late-stage, all *P*_interaction_ > 0.05) or cholecystectomy (yes vs no, all *P*_interaction_ > 0.05). Only CCL19, sTNFRI, and TRAIL retained statistical significance (*P* < 0.05) in a backward step-wise regression model including all selected inflammatory proteins, partly due to the high correlation among selected inflammatory proteins (Table 3).

**Table 3:** Spearman correlation coefficients among selected inflammatory proteins in Shanghai^a^.

	CCL19	CCL20	CRP	IL-6	sTNFRI	sTNFRII	sVEGFR3	TRAIL
CCL19	1	**0.445**	**0.315**	**0.255**	**0.404**	**0.501**	**0.318**	−0.105
CCL20		1	**0.459**	**0.504**	**0.411**	**0.548**	**0.399**	**−0.487**
CRP			1	**0.61**	**0.574**	**0.445**	**0.25**	**−0.314**
IL-6				1	**0.57**	**0.398**	**0.221**	**−0.373**
sTNFRI					1	**0.749**	**0.387**	**−0.401**
sTNFRII						1	**0.362**	**−0.436**
sVEGFR3							1	**−0.303**
TRAIL								1

^a^Correlations with P value < 0.05 are bolded.

In sensitivity analyses restricted to patients with blood collected before cholecystectomy and patients with blood collection no more than one month after diagnosis, we did not detect any appreciable changes in the magnitude of associations (Supplementary Tables 4 and 5).

Of eight proteins associated with overall survival among GBC patients in the Shanghai study, seven had data available in the Chile study. We found that elevated levels of CCL20 (*P*_trend_ across marker categories = 0.006), CRP (*P*_trend_ = 0.033), sTNFRI (*P*_trend_ = 0.014), and sTNFRII (*P*_trend_ = 0.001) were associated with a poorer overall survival, while a higher level of TRAIL was still associated with an increased overall GBC survival (*P*_trend_ = 0.015).

## Discussion

Using multiplexed assays, we conducted the most comprehensive analysis of systemic inflammatory proteins and GBC survival to date. We identified eight proteins that are associated with overall survival of patients with GBC after correcting for multiple testing. Seven proteins were associated with poorer survival, and one was associated with improved survival. Patients in the highest quartile of sTNFRII had a notably higher hazard for death compared with patients in the lowest quartile. On the other hand, patients in the highest quartile of TRAIL levels had a notably lower hazard for death compared with patients in the lowest quartile, and median survival times were ~8 months longer in patients in the highest quartile compared with patients in the lowest quartile. These results go beyond previously reported single-marker associations by demonstrating that multiple inflammatory proteins are important prognostic factors in patients with GBC.

We noticed a significant reduction in median overall survival time for seven inflammatory proteins, with the difference in survival for patients in Q4 vs Q1 ranging from 4.7 to 10.1 months. The magnitude of association with overall survival for these proteins (HR for Q4 vs. Q1 = ~3) is almost as strong as the clinical stage (HR for late- vs. early-stage = ~5), indicating the strong predictor ability of these proteins. Our findings on the associations with CRP and IL-6 are in line with other survival studies among GBC patients^19–21^. CRP is an acute-phase protein whose levels rapidly increase in response to most forms of inflammation. CRP has been included in the Glasgow prognostic score, which has been identified as an important prognostic factor for several malignancies, including GBC^20,31^. CRP is an immediate downstream marker of IL-6, which is considered a key molecule in the progression of the chronic inflammatory process to carcinogenesis^32^. Higher circulation levels of IL-6 are also associated with tumor differentiation, local invasion, and poorer survival among patients with GBC^21^. In addition to these two proteins, we identified five other proteins associated with poorer overall survival (CCL19, CCL20, sTNFRI, sTNFRII, and sVEGFR3), of which 3 (CCL20, sTNFRI, sTNFRII) can be replicated in a separate study. CCL19 is highly expressed in human cancer cells, and ligand CCL19 binding to CCR7 induces actin polymerization and pseudopodia formation. Studies have shown that CCL19 is associated with features of more aggressive disease, such as higher histological grade for breast cancer^33^. It can also promote tumor growth and progression through activation of CCR7^34^. Associations between poorer survival among cancer patients and elevated systemic levels CCL20 has been reported, and their roles in tumor progression and metastasis have previously been recognized^34^. sTNFRI and sTNFRII are major receptors for tumor necrosis factor-alpha (TNF- α) and can act as inhibitors of the biological activity of TNF-α when present at elevated concentrations^35^. sVEGFR3 has been shown to play an important role in lymphangiogenesis and is thought to be involved in the development of lymph node metastasis^36,37^.

Taken together, the large number of associations with inflammatory proteins may suggest inflammation has an essential role in survival among GBC patients. Therefore, controlling inflammation in these patients could potentially improve survival^38,39^. For example, downregulation of the expression of IL-6 through adjuvant therapy has been proposed as an alternative therapeutic option in addition to radiotherapy or chemotherapy in clinical studies of other cancers^10,40,41^. Whether use of anti-inflammatory agents could improve survival in patients with GBC merits further investigation.

Notably, TRAIL itself may have clinical utility. High expression of TRAIL is associated with favorable survival for cancers such as ovarian cancer and prostate cancer^42,43^. The TRAIL system is critically involved in the surveillance and elimination of some tumor cells^44^. The *in vivo* importance of loss of sensitivity to TRAIL-mediated apoptosis has been demonstrated by clinical studies that have shown a correlation between low TRAIL receptor expression, poor prognosis, and tumor recurrence^45^. In addition, TRAIL knockout mice exhibit enhanced formation of metastases^46^. To our knowledge, this is the first study to show that elevated levels of TRAIL are associated with an increased GBC survival. The levels of TRAIL were not correlated with clinical stage (*P* = 0.79). Given that the median overall survival for patients with late-stage GBC is ~4 months, the 8-month increase in survival for patients in the highest TRAIL quartile compared to patients in the lowest quartile is substantial. TRAIL has been proposed as a promising anticancer drug because it can selectively induce apoptosis in cancer cells, but not in healthy cells^47^. Our results thus highlight the potential for using a TRAIL-related anticancer drug for GBC treatment.

The present study is the largest to date to investigate the circulating levels of a broad range of inflammatory proteins associated with GBC survival. Despite the small sample size, we identified eight prognosis-associated inflammatory proteins after multiple testing correction, of which five proteins were validated in an independent study conducted in a separate population. Although these two studies were conducted in independently different periods (one in the 1990s and one in the 2010s), given that patients from Shanghai were not compared directly with patient from Chile, we do not believe that the differences in study period would have resulted in any bias in the estimated associations. Differences in storage time may affect the levels of the circulating proteins. However, there is no reason to expect this degradation to be associated with survival or stage. The Shanghai study could potentially be attenuated due to cytokine degradation over time given the longer storage time for these samples, but the associations observed in the Shanghai and Chile studies were similar, suggesting that the results are not affected by the time in storage. In addition, most circulating inflammation markers are not affected by freeze-thaw cycles^48^, which is usually correlated with storage time, further alleviating concerns over sample integrity. However, our results should be interpreted in light of several methodologic limitations. Although there is strong biologic plausibility for the association of inflammatory proteins with GBC survival, our observations need replication given the large number of proteins evaluated with the potential for false positives. In addition, we used overall mortality data in our analyses, as opposed to GBC-specific mortality. Given that GBC is a highly lethal malignancy with overall cure rates of ~15%, however, overall mortality is likely a good surrogate for cancer-specific mortality. Clinical stage information was incomplete for the replication site, which may have introduced some bias due to uncontrolled confounding. Finally, the study populations used for the discovery and replication phases were ethnically heterogenous. It is unclear whether factors affecting overall survival would be expected to differ by ethnicity, but most associations replicated despite this heterogeneity, highlighting the robustness of our findings.

In conclusion, our study provides epidemiologic evidence for the association of acute-phase proteins, pro-inflammatory cytokines, chemokines, and growth factors with overall survival in GBC patients, pointing to a need for further evaluation of the role of inflammatory pathways preceding GBC and their involvement in disease severity and progression. Particularly, higher levels of TRAIL were independently associated with an improvement in survival among patients with GBC. TRAIL therefore merit further investigation. Nevertheless, because our data are observational, further studies among patients with GBC are required, including placebo-controlled trials of TRAIL-related anticancer drug or anti-inflammatory agents as adjuncts to other routine therapies.

## Electronic supplementary material

Supplementary Materials
