# Author Correction: Circulating Levels of Inflammatory Proteins and Survival in Patients with Gallbladder Cancer

**DOI:** 10.1038/s41598-020-59761-2

**Published:** 2020-02-11

**Authors:** Zhiwei Liu, Troy J. Kemp, Yu-Tang Gao, Amanda Corbel, Emma E. McGee, Juan Carlos Roa, Bingsheng Wang, Juan Carlos Araya, Ming-Chang Shen, Asif Rashid, Ann W. Hsing, Allan Hildesheim, Catterina Ferreccio, Ruth M. Pfeiffer, Ligia A. Pinto, Jill Koshiol

**Affiliations:** 1 Infections and Immunoepidemiology Branch of the Division of Cancer Epidemiology and Genetics, National Cancer Institute, Bethesda, Maryland USA; 2 HPV Immunology Laboratory, Frederick National Laboratory for Cancer Research, Leidos, Biomedical Research, Inc, Frederick, MD USA; 3 Department of Epidemiology, Shanghai Cancer Institute, Shanghai, China; 4 School of Medicine, Pontificia Universidad Católica de Chile, Santiago, Chile; 5 Advanced Center for Chronic Diseases (ACCDiS), FONDAP, Santiago, Chile; 6 Department of General Surgery, Zhongshan Hospital, School of Medicine, Fudan University, Shanghai, China; 7 Hospital Dr. Hernan Henríquez Aravena, Temuco, Chile; 8 Anatomic Pathology Department, Medicine Faculty, Universidad de La Frontera, Temuco, Chile; 9 Department of Pathology, Shanghai Cancer Center, Fudan University, Shanghai, China; 10 Department of Pathology, The University of Texas MD Anderson Cancer Center, Houston, TX USA; 11 Stanford Cancer Institute, Palo Alto, CA USA; 12 Department of Health Research and Policy, Stanford School of Medicine, Palo Alto, CA USA; 13 Biostatistics Branch, Division of Cancer Epidemiology and Genetics, National Cancer Institute, Bethesda, MD USA

Correction to: *Scientific Reports* 10.1038/s41598-018-23848-8, published online 04 April 2018

This Article contains errors in the Results section.

“The median stage-adjusted survival time was 7.9 months longer in patients with the highest levels of TRAIL compared to those with the lowest levels (Table 2).”

should read:

“The median stage-adjusted survival time was 7.1 months longer in patients with the highest levels of TRAIL compared to those with the lowest levels (Table 2).”

In addition, this Article contains errors in Table 1, where the reported numbers of patients under the ‘Regular Aspirin use’ subheading are incorrect. The correct totals for ‘No’ are 32 (100.0) and 97 (96.0) under Early-stage and Late-Stage respectively, and for ‘Yes’ the correct totals are 0 (0.0) and 4(4.0) respectively.

