# Supplementary material for: Circulating Levels of Inflammatory Proteins and Survival in Patients with Gallbladder Cancer
Source: Sci Rep. 2018 Apr 4;8:5671. doi: 10.1038/s41598-018-23848-8 (PMC5884817; doi:10.1038/s41598-018-23848-8)
Supplement: Supplementary file 1 — Supplementary Materials [file 41598_2018_23848_MOESM1_ESM.pdf]

Circulating Levels of Inflammatory Proteins and Survival in Patients with Gallbladder Cancer

Zhiwei Liu [1 *], Troy J. Kemp [2], Yu-Tang Gao [3], Amanda Corbel [1], Emma E. McGee [1], Juan Carlos Roa [4,5], Bingsheng Wang [6], Juan Carlos Araya [7,8], Ming-Chang Shen [9], Asif Rashid [10], Ann W. Hsing [11,12], Allan Hildesheim [1], Catterina Ferreccio [4,5], Ruth M. Pfeiffer [13], Ligia A. Pinto [2], Jill Koshiol [1]

Short title: Inflammatory Proteins and Survival

Author Affiliations:

1. Infections and Immunoepidemiology Branch of the Division of Cancer Epidemiology and Genetics, National Cancer Institute, Bethesda, Maryland, USA
2. HPV Immunology Laboratory, Frederick National Laboratory for Cancer Research, Leidos, Biomedical Research, Inc, Frederick, MD, USA
3. Department of Epidemiology, Shanghai Cancer Institute, Shanghai, China
4. School of Medicine, Pontificia Universidad Católica de Chile, Santiago, Chile
5. Advanced Center for Chronic Diseases (ACCDiS), FONDAP, Santiago, Chile
6. Department of General Surgery, Zhongshan Hospital, School of Medicine, Fudan University, Shanghai, China
7. Hospital Dr. Hernan Henríquez Aravena, Temuco, Chile
8. Anatomic Pathology Department, Medicine Faculty, Universidad de La Frontera, Temuco, Chile
9. Department of Pathology, Shanghai Cancer Center, Fudan University, Shanghai, China

10. Department of Pathology, The University of Texas MD Anderson Cancer Center, Houston, TX, USA
11. Stanford Cancer Institute, Palo Alto, CA, USA
12. Department of Health Research and Policy, Stanford School of Medicine, Palo Alto, CA, USA
13. Biostatistics Branch, Division of Cancer Epidemiology and Genetics, National Cancer Institute, MD, USA

Corresponding author:

Zhiwei Liu, Ph.D., Infections and Immunoepidemiology Branch, Division of Cancer Epidemiology and Genetics, National Cancer Institute, 9609 Medical Center Dr, Rockville, MD 20852. Phone: 240-276-6726; Fax: 240-276-7806, email address: zhiwei.liu@nih.gov

Supplementary Materials

For the Shanghai Biliary Cancer Study, 134 samples were tested across two lots; patients tested in the first lot (N=30) were described in a previous report including some proteins in the current analysis.[1] To evaluate potential lot effects, we selected serum samples from 7 GBC cases and 22 gallstone patients and blindly re-tested them in the second lot to compare results from the two different assay runs.[2] Seven proteins (fibroblast growth factor 2 [FGF2], IL-8, stem cell factor [SCF], soluble gp130 [sGP130], sIL-6R, soluble vascular endothelial growth factor receptor-2 [sVEGFR2], and thrombopoietin [TPO]) were excluded due to significant differences between the two lots.

To further evaluate reproducibility, we included blinded duplicate aliquots from 10 participants among the samples tested with lot 1, 28 participants on lot 2, and 10 participants on the high-sensitivity panel designed to measure particularly important and difficult to measure proteins that was tested on all participants in the Shanghai Biliary Cancer Study using a single lot. Samples from the Chile Biliary Cancer Study were tested prior to the development of the high-sensitivity panel. Thus, data on inflammatory proteins in this panel were not available. We estimated coefficients of variation (CVs) and intraclass correlation coefficients (ICCs) using a log-transformed general linear model, as previously described (Koshiol et al, submitted). We further excluded 11 proteins (chemokine [C-C motif] ligand 21 [CCL21], adiponectin, adipsin, lipocalin, monocyte chemotactic protein-4 [MCP-4], plasminogen activator inhibitor-1[PAI-1], resistin, stromal cell-derived factor 1A-B [SDF-1A-B], high-sensitivity fractalkine, high-sensitivity IL-5, high-sensitivity IL-21) with overall CVs > 30% and/or ICCs<0.75. MIP3A/CCL20 was measured on both lot 1 and the high-sensitivity panel. Due to a slightly better CV and ICC from the high-sensitivity panel for MIP3A/CCL20, results from lot 1 were

excluded. Inflammatory data for IL-8 tested in the high-sensitivity panel were also retained due to a high CV and ICC.

References

1. Koshiol J, Castro F, Kemp TJ, et al: Association of inflammatory and other immune markers with gallbladder cancer: Results from two independent case-control studies. Cytokine 83:217-25, 2016

2. Shiels MS, Katki HA, Hildesheim A, et al: Circulating Inflammation Markers, Risk of Lung Cancer, and Utility for Risk Stratification. J Natl Cancer Inst 107, 2015

Supplementary Table 1. Characteristics of Patients with Gallbladder Cancer by inclusion in circulating inflammatory proteins analysis in Shanghai

	Included in Analysis?		***P* value** [a]
	**Yes (N=134, %)**	**No (N=234, %)**	
**Sex**			0.146
Female	92 (68.7)	177 (75.6)	
Male	42 (31.3)	57 (24.4)	
**Age, years**			0.303
≤ 54	13 (9.7)	36 (15.4)	
55 - 65	42 (31.3)	69 (29.5)	
≥ 66	79 (59.0)	129 (55.1)	
**Clinical stage**			0.116
Early	32 (23.9)	73 (31.6)	
Late	102 (76.1)	158 (68.4)	
**Surgery**			0.768
No	58 (43.3)	105 (44.9)	
Yes	76 (56.7)	129 (55.1)	

[a] *P* values were determined using a chi-square test.

Supplementary Table 2. Associations between inflammatory proteins and mortality among patients with gallbladder cancer in Shanghai.

Inflammatory proteins	No. of deaths	No. of patients	Adjusted HRs (95% CIs) [a]
**CXCL13**			
Q1	22	34	1.00
Q2	32	33	2.35 (1.32, 4.19)
Q3	32	34	1.70 (0.91, 3.18)
Q4	28	33	2.02 (1.09, 3.75)
P trend [b]			0.074
**CCL27**			
Q1	30	34	1.00
Q2	29	33	0.94 (0.54, 1.65)
Q3	27	34	0.66 (0.37, 1.20)
Q4	28	33	0.88 (0.48, 1.58)
P trend [b]			0.476
**CXCL11**			
Q1	24	34	1.00
Q2	27	33	1.52 (0.85, 2.73)
Q3	34	34	2.11 (1.20, 3.68)
Q4	29	33	1.51 (0.85, 2.69)
P trend [b]			0.095
**CXCL6**			
Q1	23	34	1.00
Q2	31	33	2.26 (1.25, 4.10)
Q3	29	34	1.73 (0.95, 3.15)
Q4	31	33	2.62 (1.42, 4.82)
P trend [b]			0.009
**CXCL9**			
Q1	26	34	1.00
Q2	28	33	0.96 (0.55, 1.66)
Q3	31	34	1.32 (0.74, 2.34)
Q4	29	33	0.99 (0.57, 1.72)
P trend [b]			0.795
**EGF**			
Q1	30	34	1.00
Q2	26	33	0.76 (0.45, 1.29)
Q3	29	34	0.97 (0.57, 1.65)
Q4	29	33	0.73 (0.43, 1.23)
P trend [b]			0.388
**CXCL5**			
Q1	29	34	1.00

Q2	28	33	0.95 (0.55, 1.64)
Q3	29	34	0.89 (0.52, 1.51)
Q4	28	33	0.78 (0.46, 1.32)
P trend [b]			0.338
**CCL11**			
Q1	31	34	1.00
Q2	28	33	0.77 (0.44, 1.35)
Q3	27	34	0.59 (0.34, 1.01)
Q4	28	33	0.76 (0.44, 1.31)
P trend [b]			0.138
**CCL24**			
Q1	29	34	1.00
Q2	29	33	1.30 (0.77, 2.19)
Q3	30	34	1.20 (0.71, 2.02)
Q4	26	33	1.13 (0.65, 1.97)
P trend [b]			0.697
**G-CSF**			
Q1	27	34	1.00
Q2	25	33	0.73 (0.41, 1.28)
Q3	33	34	1.58 (0.91, 2.77)
Q4	29	33	1.45 (0.85, 2.50)
P trend [b]			0.053
**CXCL1,2,3**			
Q1	27	34	1.00
Q2	27	33	1.27 (0.72, 2.24)
Q3	28	34	1.00 (0.58, 1.70)
Q4	32	33	1.31 (0.77, 2.23)
P trend [b]			0.494
**GM-CSF**			
Q1	26	34	1.00
Q2	30	33	1.03 (0.60, 1.77)
Q3	31	34	1.48 (0.84, 2.59)
Q4	26	32	0.85 (0.47, 1.54)
P trend [b]			0.874
**IL-10**			
Q1	28	34	1.00
Q2	25	33	1.09 (0.62, 1.90)
Q3	33	34	1.40 (0.83, 2.38)
Q4	27	32	1.47 (0.85, 2.53)
P trend [b]			0.106
**IL-12 (p70)**			
Q1	30	34	1.00
Q2	30	33	0.98 (0.57, 1.7)

Q3	28	34	0.99 (0.58, 1.68)
Q4	25	32	0.75 (0.44, 1.30)
P trend [b]			0.324
**IL-13**			
Q1	31	34	1.00
Q2	29	33	1.41 (0.80, 2.46)
Q3	27	34	0.75 (0.44, 1.27)
Q4	26	32	1.14 (0.66, 1.97)
P trend [b]			0.714
**IL-17A**			
Q1	29	34	1.00
Q2	28	33	0.79 (0.46, 1.38)
Q3	30	34	1.46 (0.86, 2.46)
Q4	26	32	0.72 (0.41, 1.26)
P trend [b]			0.705
**IL-1B**			
Q1	31	34	1.00
Q2	28	33	0.88 (0.52, 1.49)
Q3	32	34	1.08 (0.64, 1.82)
Q4	22	32	0.68 (0.39, 1.20)
P trend [b]			0.318
**IL-23**			
Q1	31	34	1.00
Q2	28	33	1.02 (0.59, 1.76)
Q3	31	34	0.92 (0.55, 1.54)
Q4	23	32	0.59 (0.34, 1.03)
P trend [b]			0.063
**IL-4**			
Q1	28	34	1.00
Q2	29	33	0.81 (0.47, 1.38)
Q3	29	34	0.92 (0.55, 1.57)
Q4	27	32	0.71 (0.41, 1.22)
P trend [b]			0.293
**IL-7**			
Q1	28	34	1.00
Q2	27	33	0.91 (0.52, 1.59)
Q3	28	34	1.20 (0.68, 2.12)
Q4	30	32	1.19 (0.67, 2.10)
P trend [b]			0.388
**IL-8**			
Q1	22	34	1.00
Q2	28	33	1.41 (0.78, 2.55)
Q3	31	34	2.33 (1.28, 4.23)

Q4	32	32	2.23 (1.21, 4.12)
P trend [b]			0.003
**ICAM-1**			
Q1	21	34	1.00
Q2	30	33	2.71 (1.50, 4.88)
Q3	32	34	3.44 (1.88, 6.32)
Q4	30	32	2.63 (1.42, 4.89)
P trend [b]			0.002
**CCL3**			
Q1	23	34	1.00
Q2	32	33	2.75 (1.56, 4.85)
Q3	27	34	1.63 (0.90, 2.95)
Q4	31	32	2.22 (1.25, 3.94)
P trend [b]			0.028
**IL-16**			
Q1	29	34	1.00
Q2	28	33	0.72 (0.42, 1.23)
Q3	28	34	0.62 (0.36, 1.08)
Q4	29	33	1.11 (0.65, 1.89)
P trend [b]			0.889
**IL-29**			
Q1	29	34	1.00
Q2	27	33	0.82 (0.48, 1.41)
Q3	29	34	0.84 (0.49, 1.45)
Q4	29	33	0.94 (0.54, 1.63)
P trend [b]			0.889
**IL-33**			
Q1	29	34	1.00
Q2	28	33	0.70 (0.39, 1.25)
Q3	29	34	0.77 (0.43, 1.36)
Q4	28	33	0.67 (0.36, 1.22)
P trend [b]			0.287
**CXCL10**			
Q1	27	34	1.00
Q2	27	33	1.03 (0.60, 1.78)
Q3	28	34	1.06 (0.61, 1.85)
Q4	32	33	1.46 (0.83, 2.57)
P trend [b]			0.207
**CCL2**			
Q1	26	34	1.00
Q2	27	33	1.11 (0.63, 1.97)
Q3	29	34	0.84 (0.48, 1.45)
Q4	32	33	1.64 (0.94, 2.87)

P trend [b]			0.223
**CCL8**			
Q1	30	34	1.00
Q2	27	33	0.54 (0.31, 0.93)
Q3	29	34	0.65 (0.38, 1.12)
Q4	28	33	0.63 (0.36, 1.07)
P trend [b]			0.214
**CCL22**			
Q1	29	34	1.00
Q2	31	33	1.25 (0.74, 2.12)
Q3	30	34	1.42 (0.84, 2.40)
Q4	24	33	0.51 (0.29, 0.90)
P trend [b]			0.034
**CCL4**			
Q1	24	34	1.00
Q2	25	33	1.03 (0.58, 1.81)
Q3	34	34	1.64 (0.95, 2.84)
Q4	31	33	1.55 (0.89, 2.71)
P trend [b]			0.044
**CCL15**			
Q1	23	34	1.00
Q2	31	33	1.44 (0.80, 2.60)
Q3	28	34	0.99 (0.54, 1.82)
Q4	32	33	2.20 (1.19, 4.06)
P trend [b]			0.053
**SAA**			
Q1	22	34	1.00
Q2	29	33	1.31 (0.74, 2.30)
Q3	34	34	2.01 (1.13, 3.58)
Q4	28	32	1.59 (0.89, 2.84)
P trend [b]			0.055
**sEGFR**			
Q1	30	34	1.00
Q2	25	33	0.68 (0.37, 1.23)
Q3	32	34	1.02 (0.56, 1.84)
Q4	26	31	0.83 (0.47, 1.47)
P trend [b]			0.884
**sILR-II**			
Q1	23	34	1.00
Q2	29	33	1.32 (0.75, 2.32)
Q3	33	34	1.82 (1.01, 3.28)
Q4	28	31	2.38 (1.33, 4.25)
P trend [b]			0.002

**sIL-4R**			
Q1	29	34	1.00
Q2	30	33	1.15 (0.67, 1.97)
Q3	31	34	1.78 (1.03, 3.07)
Q4	23	31	0.94 (0.54, 1.65)
P trend [b]			0.810
**CCL17**			
Q1	29	34	1.00
Q2	29	33	0.84 (0.48, 1.45)
Q3	26	34	0.81 (0.47, 1.40)
Q4	30	33	0.78 (0.46, 1.32)
P trend [b]			0.368
**TNF-a**			
Q1	25	34	1.00
Q2	28	33	1.51 (0.85, 2.66)
Q3	28	34	1.21 (0.68, 2.14)
Q4	33	33	2.57 (1.43, 4.63)
P trend [b]			0.008
**TSLP**			
Q1	30	34	1.00
Q2	27	33	0.72 (0.41, 1.27)
Q3	31	34	1.02 (0.59, 1.76)
Q4	26	33	0.69 (0.39, 1.22)
P trend [b]			0.412
**VCAM-1**			
Q1	24	34	1.00
Q2	30	33	1.75 (0.99, 3.11)
Q3	31	34	2.41 (1.34, 4.36)
Q4	28	32	2.10 (1.15, 3.83)
P trend [b]			0.010
**VEGF**			
Q1	25	34	1.00
Q2	27	33	0.84 (0.47, 1.47)
Q3	30	34	1.55 (0.90, 2.69)
Q4	32	33	1.66 (0.97, 2.87)
P trend [b]			0.012

[a] Adjusted for age groups (≤54, 55-65, or ≥66 years), sex, clinical stage (early or late), and ever had cholecystectomy, stratified by lot.

[b] Two-sided P values for trend across protein categories were assessed with the Wald test using log-transformed values of the proteins with 1 degree of freedom.

Supplementary Table 3. Inflammatory proteins in relation to clinical stage in patients with Gallbladder Cancer in Shanghai.

Inflammatory proteins	Clinical stage		*P* value [a]
	Early stage (N=32, %)	Late stage (N=102, %)	
**CCL19**			0.050
Q1	14 (43.7)	20 (19.6)	
Q2	5 (15.6)	28 (27.4)	
Q3	6 (18.8)	28 (27.5)	
Q4	7 (21.9)	26 (25.5)	
**CCL20**			0.104
Q1	10 (31.2)	24 (23.5)	
Q2	11 (34.4)	22 (21.6)	
Q3	8 (25.0)	26 (25.5)	
Q4	3 (9.4)	30 (29.4)	
**CRP**			
Q1	15 (46.9)	19 (18.6)	
Q2	6 (18.7)	27 (26.5)	
Q3	7 (21.9)	27 (26.5)	
Q4	4 (12.5)	28 (27.4)	
**IL-6**			0.019
Q1	15 (46.9)	19 (18.6)	
Q2	6 (18.7)	27 (26.4)	
Q3	7 (21.9)	27 (26.5)	
Q4	4 (12.5)	28 (27.4)	
**sTNFRI**			0.052
Q1	10 (31.2)	24 (23.5)	
Q2	12 (37.5)	21 (20.6)	
Q3	6 (18.7)	28 (27.4)	
Q4	3 (9.4)	28 (27.4)	
**sTNFRII**			0.011
Q1	15 (46.9)	19 (18.6)	
Q2	5 (15.6)	28 (27.4)	
Q3	4 (12.5)	30 (29.4)	
Q4	7 (21.9)	24 (23.5)	
**sVEGFR3**			0.829
Q1	7 (21.9)	27 (26.5)	
Q2	9 (28.1)	24 (23.5)	
Q3	9 (28.1)	25 (24.5)	
Q4	6 (18.7)	25 (24.5)	
**TRAIL**			0.786
Q1	6 (18.7)	28 (27.4)	
Q2	8 (25.0)	25 (24.5)	
Q3	9 (28.1)	25 (24.5)	
Q4	9 (28.1)	24 (23.5)	

[a] *P* values were determined using a chi-square test.

Supplementary Table 4. Associations between inflammatory proteins and mortality among patients with gallbladder cancer in Shanghai, restricted to patients with sample collected no more than one month after diagnosis (N=108).

**Inflammatory proteins**	**No. of deaths**	**No. of patients**	**Adjusted HRs (95% CIs)** [a]
**CCL19**			
Q1	19	30	1.00
Q2	20	24	1.41 (0.71, 2.83)
Q3	28	30	1.85 (0.95, 3.60)
Q4	23	24	2.62 (1.34, 5.13)
P trend [b]			0.003
**CCL20**			
Q1	17	26	1.00
Q2	24	28	2.30 (1.15, 4.59)
Q3	23	26	3.01 (1.46, 6.17)
Q4	26	28	2.71 (1.35, 5.43)
P trend [b]			0.006
**CRP**			
Q1	17	28	1.00
Q2	23	27	1.47 (0.72, 3.00)
Q3	27	27	2.28 (1.09, 4.77)
Q4	23	26	2.58 (1.28, 5.18)
P trend [b]			0.004
**IL-6**			
Q1	17	27	1.00
Q2	22	24	1.57 (0.80, 3.09)
Q3	23	28	1.24 (0.62, 2.52)
Q4	28	29	2.44 (1.23, 4.84)
P trend [b]			0.023
**sTNFRI**			
Q1	20	27	1.00
Q2	19	27	1.24 (0.64, 2.38)
Q3	30	30	3.37 (1.85, 6.14)
Q4	21	23	3.03 (1.52, 6.06)
P trend [b]			0.0002
**sTNFRII**			
Q1	19	29	1.00
Q2	25	29	1.01 (0.51, 2.00)
Q3	23	25	1.78 (0.88, 3.60)
Q4	23	24	3.42 (1.65, 7.12)
P trend [b]			0.00005
**sVEGFR3**			
Q1	20	27	1.00

Q2	21	26	2.73 (1.40, 5.30)
Q3	27	28	2.44 (1.29, 4.64)
Q4	22	26	2.27 (1.15, 4.49)
P trend [b]			0.023
**TRAIL**			
Q1	27	29	1.00
Q2	21	26	0.47 (0.25, 0.89)
Q3	26	30	0.56 (0.31, 1.01)
Q4	16	23	0.25 (0.12, 0.50)
P trend [b]			0.0004

[a] Adjusted for age groups (≤54, 55-65, or ≥66 years), sex, clinical stage (early or late), and ever had cholecystectomy, stratified by lot.

[b] Two-sided P values for trend across protein categories were assessed with the Wald test using log-transformed values of the proteins with 1 degree of freedom

Supplementary Table 5. Associations between inflammatory proteins and mortality among patients with gallbladder cancer in Shanghai, restricted to patients with sample collected before any therapies (N=130).

Inflammatory proteins	No. of deaths	No. of patients	Adjusted HRs (95% CIs) [a]
**CCL19**			
Q1	22	33	1.00
Q2	29	33	1.50 (0.82, 2.74)
Q3	31	33	2.17 (1.16, 4.04)
Q4	29	31	3.27 (1.77, 6.03)
P trend [b]			6.9×10^{-5}
**CCL20**			
Q1	25	34	1.00
Q2	28	33	1.81 (1.04, 3.15)
Q3	29	32	2.83 (1.62, 4.96)
Q4	29	31	2.69 (1.50, 4.82)
P trend [b]			1.8×10^{-4}
**CRP**			
Q1	22	33	1.00
Q2	28	32	1.86 (1.02, 3.41)
Q3	31	32	2.60 (1.40, 4.83)
Q4	29	32	2.85 (1.55, 5.21)
P trend [b]			3.6×10^{-4}
**IL-6**			
Q1	22	33	1.00
Q2	31	33	1.69 (0.94, 3.05)
Q3	28	33	1.74 (0.95, 3.19)
Q4	29	30	3.29 (1.80, 6.03)
P trend [b]			2.5×10^{-4}
**sTNFRI**			
Q1	26	34	1.00
Q2	24	32	1.41 (0.79, 2.49)
Q3	32	32	4.46 (2.55, 7.79)
Q4	28	30	2.70 (1.51, 4.84)
P trend [b]			9.1×10^{-6}
**sTNFRII**			
Q1	23	33	1.00
Q2	28	32	1.36 (0.74, 2.50)
Q3	31	34	2.40 (1.31, 4.38)
Q4	28	29	4.29 (2.21, 8.35)
P trend b			2.4×10^{-6}
**sVEGFR3**			
Q1	26	34	1.00
Q2	27	33	2.60 (1.44, 4.72)

Q3	33	33	2.84 (1.63, 4.96)
Q4	24	28	2.96 (1.57, 5.59)
P trend [b]			3.2×10^{-4}
**TRAIL**			
Q1	30	32	1.00
Q2	27	32	0.44 (0.25, 0.77)
Q3	30	33	0.58 (0.34, 1.00)
Q4	24	33	0.24 (0.13, 0.44)
P trend [b]			4.9×10^{-5}

[a] Adjusted for age groups (≤54, 55-65, or ≥66 years), sex, clinical stage (early or late), and ever had cholecystectomy, stratified by lot.

[b] Two-sided P values for trend across protein categories were assessed with the Wald test using log-transformed values of the proteins with 1 degree of freedom

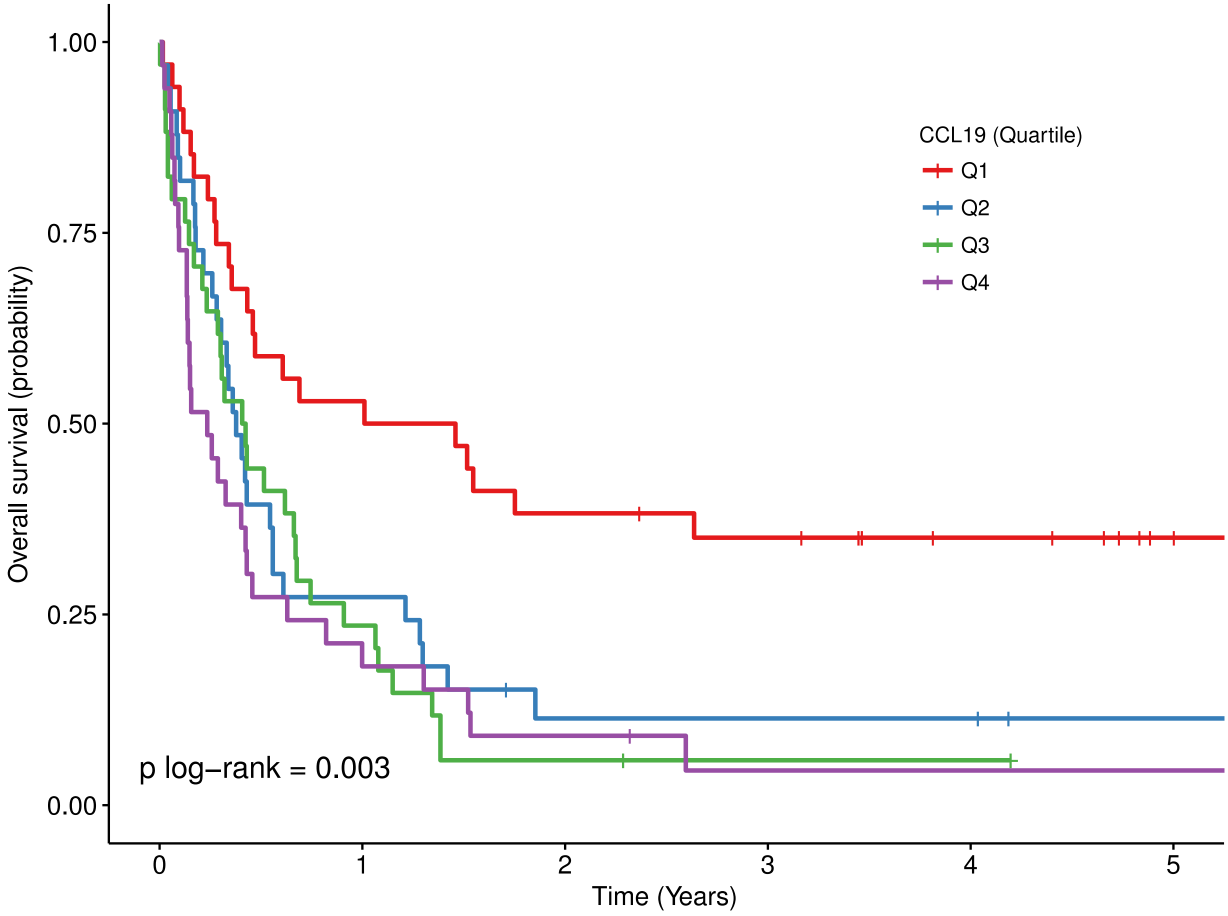

Number at risk

CCL19 (Quartile)	0	1	2	3	4	5
Q1	34	18	13	11	7	2
Q2	33	9	3	3	3	1
Q3	34	8	2	1	1	0
Q4	33	6	3	1	1	1

Supplementary Figure 1. Kaplan-Meier survival estimates for patients with GBC. Overall survival curves of evaluated patients stratified by circulating CCL19 levels (quartile).

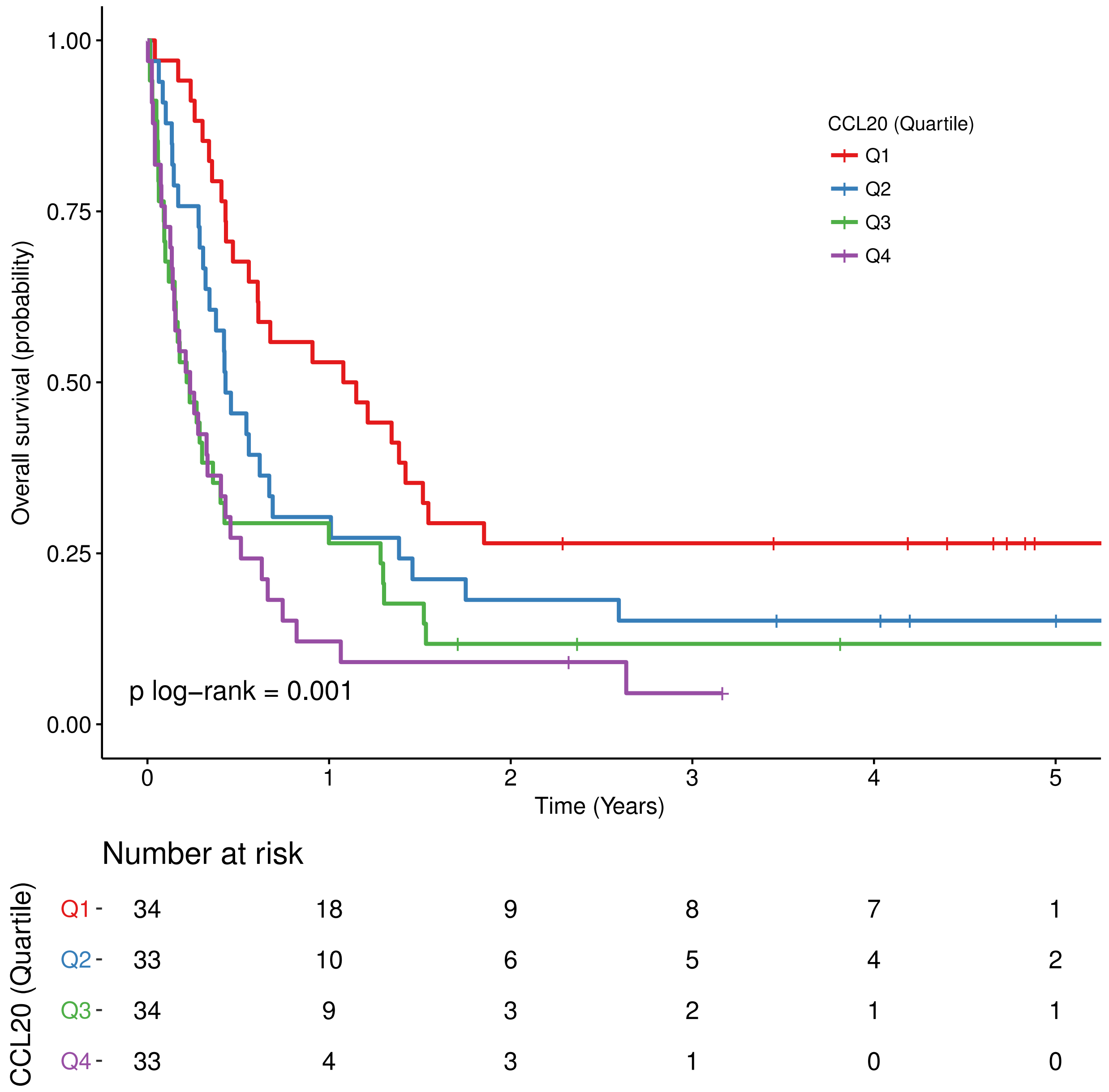

Supplementary Figure 2. Kaplan-Meier survival estimates for patients with GBC. Overall survival curves of evaluated patients stratified by circulating CCL20 levels (quartile).

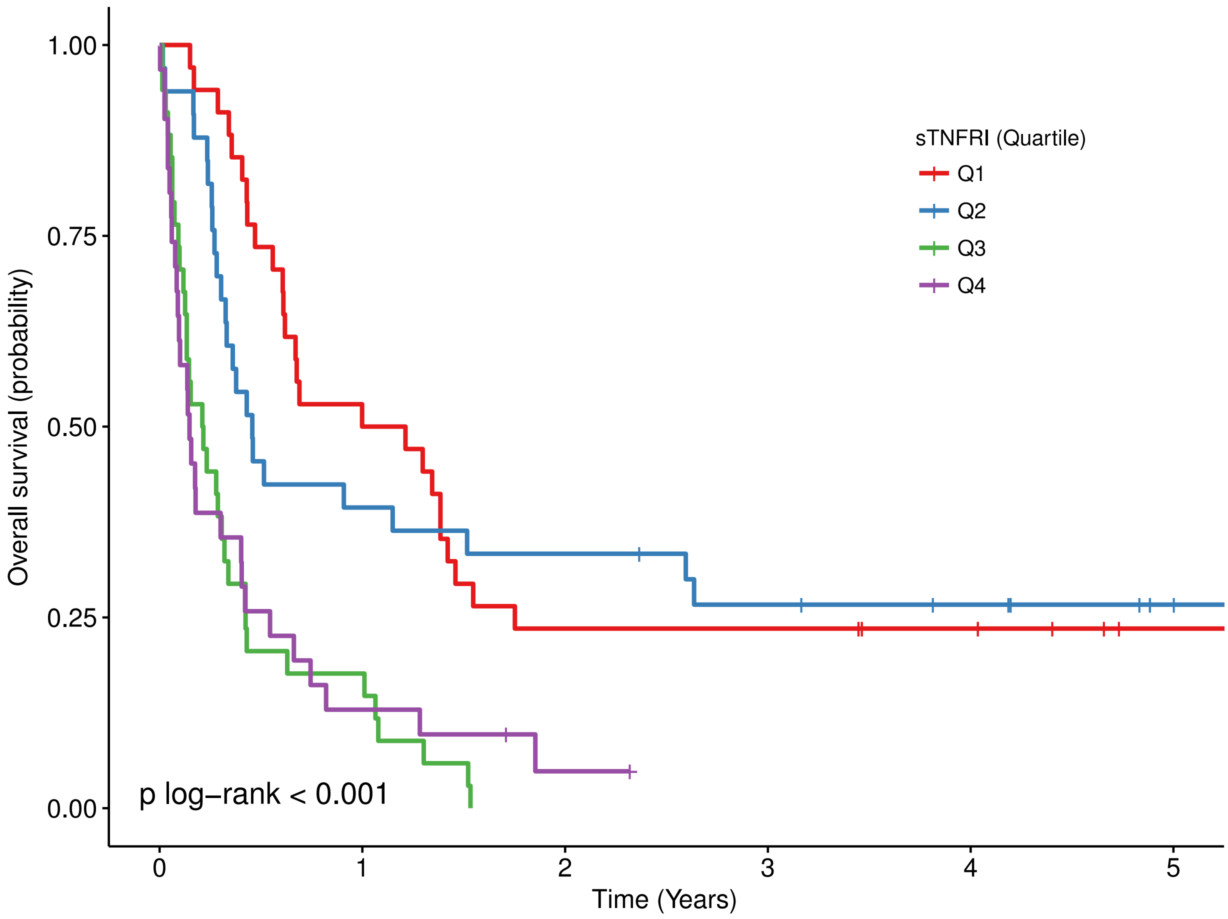

Number at risk

sTNFRI (Quartile)	0	1	2	3	4	5
Q1	34	17	8	8	6	2
Q2	33	13	11	8	6	2
Q3	34	6	0	0	0	0
Q4	31	4	1	0	0	0

Supplementary Figure 3. Kaplan-Meier survival estimates for patients with GBC. Overall survival curves of evaluated patients stratified by circulating sTNFRI levels (quartile).

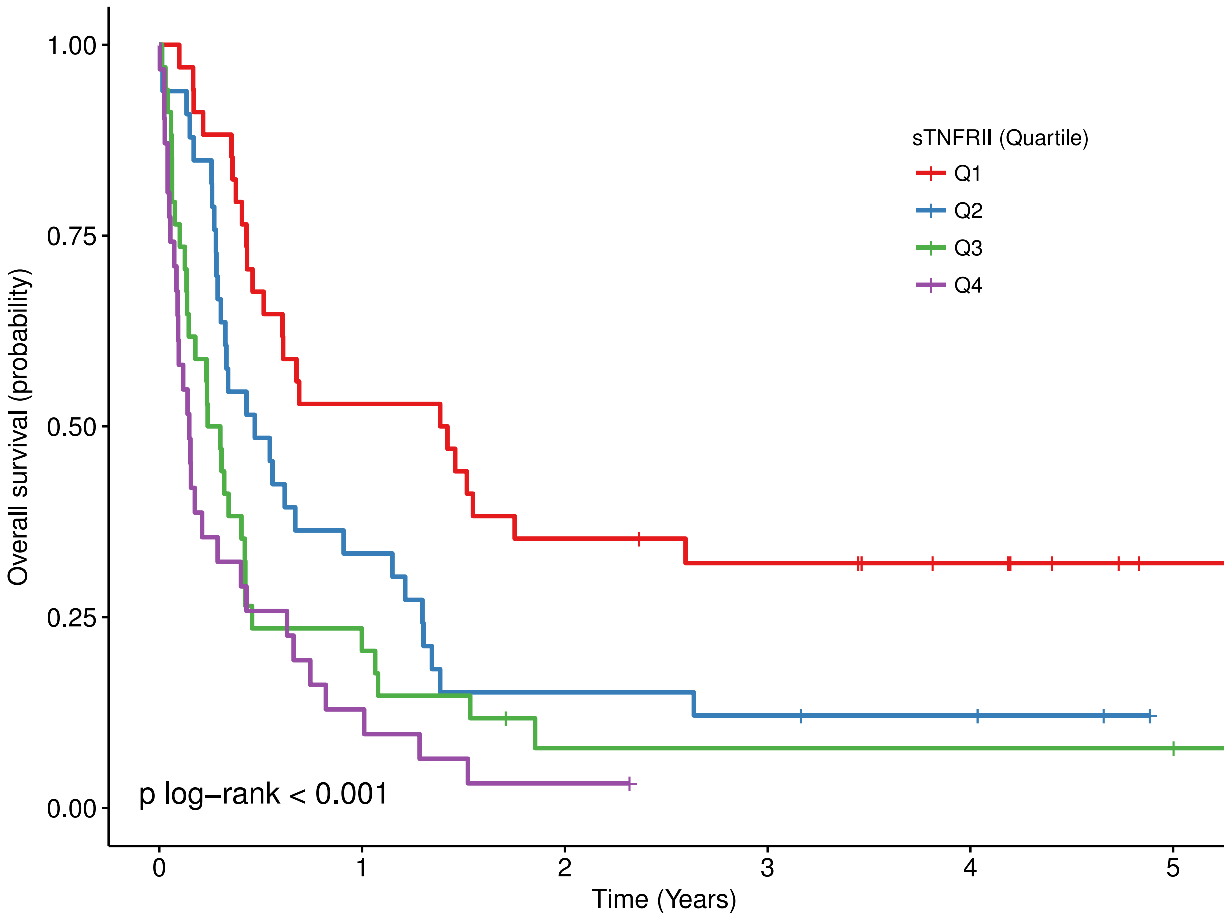

Number at risk

sTNFRII (Quartile)	0	1	2	3	4	5
Q1	34	18	12	10	7	2
Q2	33	11	5	4	3	0
Q3	34	7	2	2	2	2
Q4	31	4	1	0	0	0

Supplementary Figure 4. Kaplan-Meier survival estimates for patients with GBC. Overall survival curves of evaluated patients stratified by circulating sTNFRII levels (quartile).

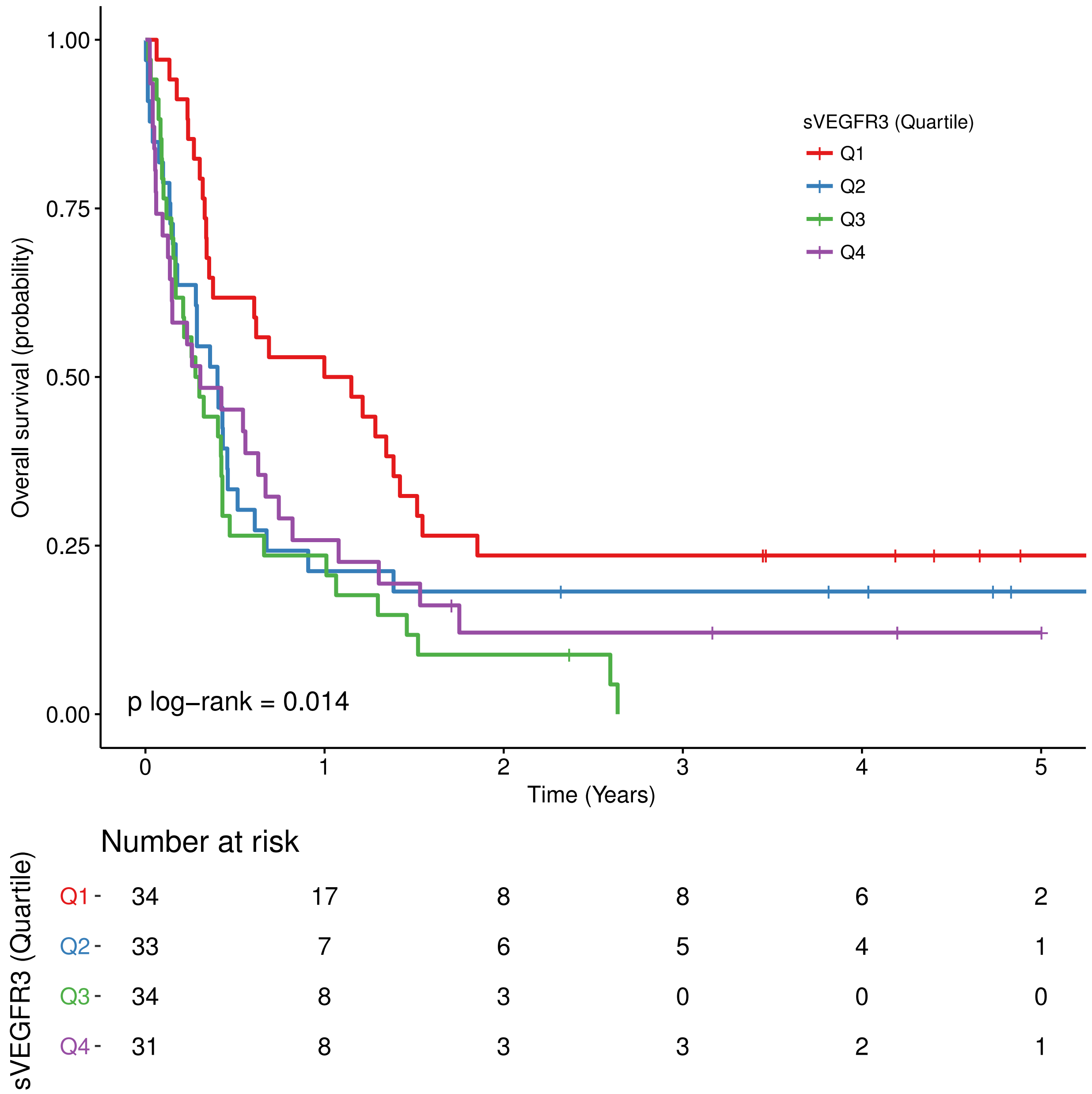

Supplementary Figure 5. Kaplan-Meier survival estimates for patients with GBC. Overall survival curves of evaluated patients stratified by circulating sVEGFR3 levels (quartile).
